# Serum biomarkers in interstitial lung diseases

**DOI:** 10.1186/1465-9921-6-78

**Published:** 2005-07-21

**Authors:** Argyris Tzouvelekis, George Kouliatsis, Stavros Anevlavis, Demosthenes Bouros

**Affiliations:** 1 Interstitial Lung Disease Unit, Royal Brompton Hospital, Imperial College, Faculty of Medicine, London, UK; 2 Department of Pneumonology, Medical School, Democritus University of Thrace, Greece

**Keywords:** Serum biomarkers, interstitial lung diseases, idiopathic pulmonary fibrosis, systemic sclerosis, sarcoidosis, KL-6, surfactant proteins, cytokines

## Abstract

The use of biomarkers in medicine lies in their ability to detect disease and support diagnostic and therapeutic decisions. New research and novel understanding of the molecular basis of the disease reveals an abundance of exciting new biomarkers who present a promise for use in the everyday clinical practice. The past fifteen years have seen the emergence of numerous clinical applications of several new molecules as biologic markers in the research field relevant to interstitial lung diseases (translational research). The scope of this review is to summarize the current state of knowledge about serum biomarkers in interstitial lung diseases and their potential value as prognostic and diagnostic tools and present some of the future perspectives and challenges.

## Introduction

The use of biomarkers in medicine lies in their ability to detect disease and support diagnostic and therapeutic decisions. New research and novel understanding of the molecular basis of the disease reveals an abundance of exciting new biomarkers who present a promise for use in the everyday clinical practice. The initial evaluation of a serum biomarker concerns its expression in patients with the disease and in normal individuals in order to define sensitivity and specificity. The sensitivity of a test is defined as the proportion of patients with disease having a positive test whereas the specificity is the proportion of patients without the disease who have a negative or normal test (Table 1). Consequently the serum level of an ideal marker should: 1) increase pathologically in the presence of the disease (high sensitivity), 2) not increase in the absence of the disease (high specificity), 3) add information about the risk or prognosis 4) change in accordance with the clinical evolution, reflecting the current status of disease, or better 5) anticipate clinical changes, i.e. indicating the presence of relapse before it becomes obvious at a clinical level and finally 6) relate to disease burden and extent 7) be reproducible (as determined by the low coefficient of variation), 8) be of easy and cheap determination [1,2].

**Table 1:** Definition of sensitivity, specificity, and predictive values of a discrete test for the presence of a disease

**Sensitivity, Specificity, and Predictive Value of a Discrete Test for the Presence of a Disease**		
	**Disease Present**	**Disease Absent**
**Positive Test**	a (true positive)	b (false positive)
**Negative Test**	c (false negative)	d (true negative)





Very few markers present a threshold at which the risk suddenly rises. The interplay between sensitivity and specificity and the nature of the disease under prediction assigns suitable cut-off points. Sensitivity and specificity calculated at various cut-off points give rise to a receiver-operating-characteristic (ROC) curve [2]. A clinically useful biomarker will be one with the largest area under the ROC curve. A number of novel blood biomarkers of lung disease including cytokines, enzymes, adhesion molecules, collagen relevant products and products of type II epithelial cells, have been studied for their clinical applicability.

### The need for a biomarker in Interstitial Lung Diseases

In diffuse lung disease, there has been confusion for decades because of the term "disease activity" that variably means a) inflammation and responsiveness to therapy; and b) progressiveness. In diffuse lung disease, there are two separate therapeutic goals: a) a short term response as judged by improvements in pulmonary function test (PFT) and regression of imaging abnormalities as judged mainly by high resolution computed tomography (HRCT); and b) slowing or prevention of decline. It follows logically that a useful biomarker might conceivably predict either responsiveness (i.e. the inflammatory component) or the risk of progression of fibrotic disease. Therefore, the clinical utility of a biomarker is critically dependant upon whether clinicians have tools in diffuse lung disease that accurately predict: a) responsiveness and b) progression of disease. There is much less need to predict responsiveness than progressiveness. HRCT is reasonably accurate in the separation between a group of patients in which disease is clearly irreversible and a group of patients in whom responsiveness is reasonably likely [3]. Hunninghake et al. [4,5] reported that a confident HRCT pattern diagnostic of UIP can predict outcome as well as a histologic diagnosis of usual interstitial pneumonia (UIP). They also concluded that, when a HRCT pattern diagnostic of UIP is present, the extent of disease is the best predictor of mortality. However, the change in extent of disease is not yet confirmed to be predictive of survival. Moreover, even in cases in which HRCT is indeterminate in this regard the clinician has an answer very quickly with a trial of corticosteroids. By contrast, there is no reliable routine tool with which to predict the likelihood of progression of fibrotic disease with or without therapy. Even in the usually progressive disorder, (UIP), there is significant heterogeneity in the risk of progression and in other disorders. HRCT may be quite good at distinguishing between individual disorders but it does not have any track-record at all in predicting the rapidity of decline, within individual diseases. Moreover, the value of bronchoalveolar lavage (BAL) in assessment of prognosis and treatment in the strictly defined UIP subset is still an open question [6]. Thereby, clinicians need a biomarker with which to predict decline; a fibrogenetic biomarker.

Another key benefit a biomarker might provide is more accurate prognostic information from change in that biomarker, especially if the information can be obtained rapidly (i.e. short term change with treatment). The current serial tests including serial HRCT and PFT provide scattered statements regarding the prediction of long-term outcome. Flaherty et al. [7] stated that short term changes in baseline PFT are strongly predictive of long term survival in patients with well defined UIP and non-specific interstitial pneumonia (NSIP), whereas serial changes in HRCT were of limited value. In addition, Latsi et al. [8] have recently demonstrated that serial PFT is probably the best so far for this role in UIP/ NSIP patients (predicts survival and longitudinal behaviour of the disease more accurately than baseline variables) but represents only an approximate guide and you have to wait for months for the information.

### Requirements for a biomarker in Interstitial Lung Diseases

On the contrary to the listed theoretical requirements reflecting the profile of an ideal marker, a useful biomarker in the real world of clinical practice, simply needs to fulfil an unmet need. If current non-invasive and semi-invasive tools predict outcome very accurately, a biomarker will add little extra information of value. Therefore, it simply needs to provide more accurate information about progressiveness than current modalities, not independent information. Is it mandatory for a biological predictor to add independent information about prognosis? Is it necessary for a clinician to score the scan or the factor in the lung function severity mathematically, every time a biomarker is to be measured? Additionally, this information should be transferable, since the definition of interstitial lung diseases (ILDs) is not yet constant and therefore test accuracy may vary considerably from one setting to another [9].

Regarding serial monitoring, whether or not a biomarker is highly reproducible is less crucial than clear quantification of the reproducibility, in order to distinguish between true change and measurement "noise". A marker might be poorly reproducible and yet highly accurate when it does clearly change. Furthermore, a poorly reproducible biomarker may be highly accurate when the range is categorized into for example a five – point semi quantitative scale, in order to address reproducibility issues.

Moreover, there is confusion between diagnostic and prognostic requirements. A diagnostic marker needs to be sensitive and specific [10]. However, a prognostic biomarker could, in principle, be normal when disease is present but stable. Alternatively, based on the conception that the best diagnosis is prognosis, the most useful diagnostic marker will be the one that separates patients against the disease-specific outcomes [11]. Additionally, relation to disease burden and extent should not represent prerequisites for a valuable biomarker. Clinicians have PFT and HRCT for this task. Many abnormalities are simply by-products of extensive fibrotic disease and are not informative about pathogenesis/progressiveness, per se. Failure to distinguish between severity and progressiveness has severely hindered clinical research in this field. Adding knowledge about progressiveness is one of the most fruitful applications. In addition, the ideal biomarker should not be confounded by severity ("current status"). The ideal marker should normalize in value when an effective treatment is found to prevent decline of extensive fibrotic disease, even though the "current status" of disease severity is unchanged.

In conclusion, the clinical need for a biomarker in diffuse lung disease regarding the presence or absence of the disease, the histospecific diagnosis and the prediction of responsiveness has probably little value. On the other hand, clinical necessity becomes invaluable regarding the baseline prediction of rate of progression and the early prediction of progression based on serial change with treatment. This will allow anti-inflammatory or other treatments to be evaluated or eventually modified before they have failed.

The scope of this review is based on the fact that although there are numerous published papers investigating the utility of biomarkers in the clinical research field, the number of review articles summarizing the current state of knowledge about the clinical applications of these molecules as diagnostic and prognostic tools in the research field relevant to common ILDs such as idiopathic pulmonary fibrosis (IPF), scleroderma, sarcoidosis, as well as other ILDs including radiation and drug- induced pneumonitis, pediatric ILDs and occupational and environmental diseases, still remains inadequately small.

## Serum biomarkers in Interstitial Lung Diseases

Beyond other important functions, the lung epithelium produces complex secretions, including mucus blanket, surfactant proteins, as well as several proteins important for host defense [12].

Sampling the epithelial lining fluid by bronchoalveolar lavage (BAL) represents the common means of studying the proteins secreted by the lung epithelium and investigating their alterations in lung disorders [13]. However, the past fifteen years, scientists led by pioneering studies [14] that showed the presence of these proteins in the bloodstream as well, even though in small amounts, demonstrated using enzyme-linked immunosorbent assays (ELISA) significant variations of these proteins' levels in the serum of patients with different ILDs. The latter suggests that their assay might represent a novel approach in the assessment of lung diseases with still elusive pathogenesis, prognosis, diagnosis and therapeutic interventions. Because these proteins are mainly, if not exclusively secreted within the respiratory tract, their occurrence in the vascular compartment can be explained by several hypothetical mechanisms including [12]:

• Leakage from the lung into the bloodstream resulting from the increased permeability of lung vessels and the destruction of the barrier between alveolar epithelium and endothelium caused by injury to the basement membrane

• Increased production by the alveolar type II cells coupled with an increase in total type II cells per lung due to diffuse hyperplasia and

• Diminished clearance rates from the circulation.

In the present review article we have focused on recent publications concerning the most studied and interesting lung peripheral biologic markers in ILDs, namely: lung epithelium specific proteins (markers of epithelial damage), circulating cytokines estimating various types of inflammatory activity and finally enzymes and metabolites, products of epithelioid cells and derivatives of activated macrophages. (Table 2)

**Table 2:** List of studied serum biomarkers in ILDs

**Lung epithelium-specific proteins**	**Surfactant-associated proteins**
	• SP-A
	• SP-D
	**Mucin-associated antigens**
	• KL-6/MUC1
	**Clara-cell protein**
	• CC16
	**Other lung epithelial markers**
	• CK-19
	• Ca 19-9
	• SLX
**Cytokines and other serological parameters**	**Chemokines and cytokines**
	• MCP-1
	• MIP-1a
	• ITAC/CXCL-11
	• TNF
	**Anti-oxidant enzymes and collagen peptides**
	• Glutathione
	• Type III procollagen peptide
	**Markers of T-cell activation**
	• sIL-2R
	**Markers of macrophage/monocyte activity**
	• ACE
	• Neopterin
	• b-glucuronidase
	• LDH

Abbreviations: ACE: Angiotensin Converting Enzyme, Ca 19-9: Carbohydrate antigen Sialyl Lewis (a), CC16: Clara-cell protein 16, CK19: Cytokeratin fragment 19, CXCL-11: CXC chemokine 11, KL-6: Krebs von den Lungen-6, LDH: Lactate Dehydrogenase, MCP-1: Monocyte Chemoattractant Protein-1, MIP-1a: Monocyte Inflammatory Protein-1a, MUC: Mucin, ILDs: Interstitial Lung Diseases, ITAC: Interferon-inducible T cell-a chemoattractant, sIL-2R: soluble interleukin-2 receptor, SLX: Carbohydrate antigen Sialyl Lewis (x), TNF: Tumor Necrosis Factor

## Lung epithelium-specific proteins

### Surfactant-associated Proteins

Pulmonary surfactant is a complex and highly surface active material covering the alveolar space of the lung. Biochemically, surfactant is a molecular mixture composed mainly of structurally heterogeneous phospholipids. A major function of pulmonary surfactant is to reduce the surface tension at the air-liquid interface of the alveolus, thereby preventing alveolar collapse on expiration. It has also been demonstrated that the surfactant contains specific proteins [15]. Four surfactant-specific proteins with different structural and functional properties have so far been identified. They were named surfactant protein-(SP)-A, SP-B, SP-C and SP-D according to the chronologic order of their discovery [16] and have been divided in two distinctive groups, the low-molecular-weight hydrophobic SP-B and SP-C and the high-molecular-weight-hydrophilic SP-A and SP-D. The latter belong to the collectin subgroup of the C-type lectin superfamily and are produced by two types of non-ciliated epithelial cells in the peripheral airway, Clara cells and alveolar type II cells. Studies have demonstrated that these proteins play important roles in the innate immune system of the lung [17] and have been used as useful markers for confirming the diagnosis and evaluation of disease activity of various ILDs since they reflect the epithelial damage and turnover. SP-A has also been used as a marker for lung adenocarcinomas to differentiate lung adenocarcinomas from other types and metastatic cancers from other origins, and to detect metastasis of lung adenocarcinomas [18]. Even if the lung appears as the major site of their synthesis, their expression is not restricted to the respiratory tract but has been detected in several extrapulmonary tissues [19,20].

### Mucin-associated Antigens

Mucins are major components of the mucus layer covering the airway epithelium. They consist of high-molecular-weight glycoproteins belonging to a broad family of mucin peptides [12]. Mucins are either associated with membranes or secreted at the surface of the respiratory tract [12]. Krebs von den Lungen-(KL)-6 is mainly associated with cellular membranes. It was initially described by Kohno et al. [14] as a high-molecular-weight glycoprotein and was classified as human MUC1 mucin. Immunohistochemistry has mainly detected KL-6 in alveolar type II and epithelial cells of the respiratory bronchioles. Although KL-6 is predominantly expressed by airway cells, however is not entirely lung specific, since it is also present on other somatic cells, such as pancreatic cells, eosophageal cells and fundic cells of the stomach [21]. Functionally KL-6 has been demonstrated to be inducible for the migration of human lung and skin fibroblasts, suggesting a potential role in the lung fibrogenic process [22]. Additionally KL-6 is a sensitive indicator of damage to alveolar type II cells, which strongly express this mucin at their surface. Type II pneumonocytes are regenerated over the alveolar basement membrane after the death of type I pneumonocytes over the first stage of lung injury. Therefore, its raise would theoretically represent the destruction of the normal lung parenchyma and architecture, the increased permeability of the air-blood barrier as long as the regenerating process as expressed by type II pneumonocytes' activity. Towards this direction KL-6 has been reported by several studies some of them cited in this review article as a sensitive marker for ILDs such as IPF, collagen vascular disease-associated interstitial pneumonia (CVD-IP), radiation pneumonitis, hypersensitivity pneumonitis and pulmonary sarcoidosis.

### Clara Cell Protein (CC16)

The Clara Cell secretory protein-(CC)-16 is a low-molecular-weight protein of 16 kDa is secreted in large amounts into the lumen of the respiratory tract by nonciliated bronchiolar Clara cells in humans and rodents [23]. Immunohistochemical studies have shown that CC16 is not an entirely specific and exclusive product of Clara cells or even the lung [24,25]. The exact functions of CC16 are still elusive but there is increased knowledge that CC16 serves as an important immunosuppressive and anti-inflammatory mediator in the lung [12]. Additionally CC16 can inhibit production of interferon-γ (IFN-γ) by peripheral blood mononuclear cells [12]. Serum CC16 has been demonstrated to elevate in several conditions known to be related with an impairment of the air-blood barrier, including pulmonary fibrosis [26] or lung injury caused by firesmoke [27]. Thus, several group of scientists estimated CC16 blood levels to determine whether these are associated with the degree of lung involvement in ILDs and thereby can serve as a useful biomarker of the disease activity and severity.

### Other lung-epithelial markers

Cytokeratin is a specific cytoskeletal structure expressed in epithelial cells, including bronchial epithelia [28,29]. Of the 27 known subunits of cytokeratin, cytokeratin fragment 19 (CK19) has been found soluble in serum and its levels have already been evaluated as a useful tumour marker for lung cancer [30]. Since it has been suggested that CK19 is released from injured bronchial epithelium [31], the past ten years it has been hypothesized and eventually indicated by several reports in the literature that CK19 serum levels are elevated in IPF and other ILDs and can be well correlated with the disease prognosis and diagnosis. Thus, in nowadays, there is an ongoing attempt to scrutinize the value of CK19 in evaluating the severity of lung injury as reflected by the increasing number of published papers investigating the role of this biologic marker in ILDs.

The cancer-associated antigens sialyl Lewis (a) (Ca 19-9) and sialyl Lewis (x) (SLX) are carbohydrate structures used as markers of cell differentiation and embryonic development [32]. It has been reported that a variety of these antigens is expressed on the cell surface during tumor progression [33]. Elevated circulating levels of these antigens have been associated with neoplastic transformation and metastases and therefore been used as tumor markers in the diagnosis of lung adenocarcinoma [34,35]. Serum levels of carbohydrate antigens have been found, however, also raised in some patients with non-malignant lung diseases such as IPF [36], tuberculosis [37] and diffuse parabronchiolitis [38] and moreover immunohistochemical analysis demonstrated their presence in the hyperplastic bronchiolar epithelium, on the surface epithelium cells and on exudates in air space. Repeated damage to the lungs may force these antigens into the blood circulation resulting to the elevated serum levels of these markers detected in these patients. Hence, it has been speculated that their elevation could mirror the extent of lung injury and serve as a valuable prognosticator of disease progression.

## Cytokines and other serological parameters

A number of cytokines probing different aspects of the immunopathogenesis of ILDs have been tested for their clinical usefulness as serum biomarkers for monitoring disease severity and predicting response to treatment and therefore leading to early diagnosis of progressive disease and determination of therapeutic interventions.

Monocyte chemoattractant protein-(MCP)-1 and monocyte inflammatory protein- (MIP)-1a belong to the C-C subfamily of the chemokine family and appear to be important factors in the monocyte/macrophage-mediated inflammatory process in the ILDs [39,40]. It has been reported that epithelial cells, macrophages and vascular endothelial cells are the major-MCP-1-producing cells in IPF lung tissue [41]. Moreover, an elevation of MCP-1 and MIP-1a concentrations in BAL from patients with IPF and sarcoidosis has recently been demonstrated [40,42]. Tempted by the latter observation several group of scientists aimed to measure the levels of MCP-1 and MIP-1a in the serum and determine their clinical significance as a reliable and easily repeatable serological parameters for the differential diagnosis of ILDs and the monitoring of their clinical course.

IFN-inducible T cell-a chemoattractant-ITAC/CXCL-11 is a chemoattractant CXC chemokine with versatile properties including immunomodulatory, definsin-like antimicrobial, antiangiogenic and potentially antifibrotic activities. It has been reported to inhibit angiogenesis and thereby to reduce aberrant vascular remodeling resulting in diminished fibrosis [43,44]. Studies have demonstrated a direct association of this molecule with the pleiotropic cytokine IFN-γ1b [43,44] evidence that captured the interest of both clinicians and researchers to investigate the effects of this newly applied treatment on biologic markers such as ITAC/CXCL-11 associated with fibrosis, angiogenesis and immunomodulation in the plasma of patients with ILDs and ultimately reveal novel molecular pathways which support IFN-γ1b therapeutic utilities.

In addition, the activity and release of a large body of inflammatory mediators including tumor necrosis factor (TNF), antioxidant enzymes (glutathione), procollagen peptides (type III) and markers of cell damage such as lactate dehydrogenase (LDH) have been evaluated as prognostic and monitoring tools of the disease development, activity and progression in a variety of occupational and environmental studies.

Other delineated serological parameters that have been scrutinized for their clinical efficacy as serum biologic markers of the disease severity and the activation of several inflammatory cells contributing to the immunopathogenesis of the disease include markers of T-cell activation and markers for the evaluation of macrophage/monocyte activity.

Soluble IL-2 receptor (sIL-2R) represents a biomarker of the T-cell activation and can be found in BAL fluid and serum of sarcoidosis patients and it is released by activated alveolar immune cells. In addition to lymphocytes, activated sarcoid alveolar macrophages are capable of expressing increased numbers of sIL-2R upon activation [45]. There are several reports in the literature, some of them are reviewed here, that reveal an intimate relationship between this parameter and the clinical activity of the disease, providing further evidence for the close linkage between the course of sarcoidosis and the activated state of T-cells [45].

Parameters suitable for gauging the activity of the macrophage lineage in ILDs and specifically sarcoidosis and occupational diseases have been delineated and comprise the angiotensin converting enzyme (ACE) a product of epithelioid cells that reflect the granuloma burden of the entire body, neopterin a metabolite of the guanosinetriphosphate pathway that is released by activated macrophages and monocytes under the control of IFN-γ produced by T-cells [45] and b-glucuronidase a lysosomal enzyme, associated with increased phagocytic activity [46]. Serum and BALF levels of these molecules have been found elevated in patients with sarcoidosis and environmental lung disorders and have been used in the everyday clinical practice as markers for the clinical assessment and follow-up of granulomatous inflammation and dust-induced inflammatory response.

## 1. Serum biomarkers in Idiopathic Pulmonary Fibrosis and Collagen Vascular Disease – associated Interstitial Pneumonia (Tables 3 and 4)

**Table 3:** Studies measuring serum biomarkers in patients with IPF

**Investigator**	**Patients Controls**	**Biomarker / Summary**	**ROC curve analysis Cut-off values**	**Specificity – Sensitivity Diagnostic accuracy**	**Limitations**
Kobayashi et al. ^50^	45 67	KL-6: a serum marker for interstitial pneumonia	Yes KL-6: 500–550 U/ml	KL-6: 95 - 95%	Non ILD-specific marker Potential influence by malignancies / Small number of patients
Takahashi et al. ^56^	52 108	Serum SP-A and SP-D as prognostic factors IPF and their relationship to disease extent	Yes SP-A: 45 ng/ml SP-D: 110 ng/ml	SP-A: 79 - 94% SP-D: 85 - 95%	Weak correlations with CT features / Inconclusive analysis of disease mortality / Reproducibility issues / Small number of patients
Yokoyama et al. ^57^	14	Circulating KL-6 predicts the outcome of rapidly progressive IPF	No	Not estimated	Small number of patients / Outcome prediction with pretreatment levels / Non ILD-specific marker
Ishii et al. ^61^	49 9	High serum concentrations of SP-A in UIP compared with NSIP	No	Not estimated	Small number of patients / Overlap of SP-A levels in UIP and NSIP / No ROC curve analysis / cut-off levels
Satoh et al. ^69^	41	Ca 19-9 serum levels as markers of disease activity in patients with fibrosing lung disease	No	Not estimated	Small number of IPF patients / No serial measurement / No ROC curve analysis / cut-off levels
Takayama et al. ^70^	16	Elevated Ca 19-9 serum levels are well Correlated with the disease activity	No	Not estimated	Small number of IPF patients / No serial measurement / No ROC curve analysis / cut-off levels
Yokoyama et al. ^71^	35 70	Superiority of KL-6 serum levels as a diagnostic marker of interstitial pneumonia	Yes KL-6: 449 U/ml Ca 19-9: 26 U/ml SLX: 41 U/ml	KL-6: 74 - 91 - 99% Ca 19-9: 43 - 77 - 94% SLX: 20 - 71 - 96%	Small number of IPF patients / Non ILD-specific markers / Not definitive cut-off values
Satoh et al. ^72^	23	Western blotting of serum SLX may serve as differentiator between lung adenocarcinoma and IPF	Yes SLX: 50 U/ml	SLX: 93 - 83%	Small number of patients / Low statistical power / Technical deficiencies
Suga et al. ^74^	86 10	Clinical significance of MCP-1 levels in BAL and serum in patients with ILDs	No	Not estimated	No definitive relation with disease severity / Influence by steroid treatment / Potential influence by other lung disorders
Strieter et al. ^75^	32	Effects of IFN-γ-1b on biomarker expression in patients with IPF	No	Not estimated	Pre- and post-treatment fluctuations / Not definitive results

Abbreviations: BAL: Bronchoalveolar lavage, Ca 19-9: Carbohydrate antigen Sialyl Lewis (a), CT: Computed Tomography, IFN-γ: Interferon-γ, ILD: Interstitial Lung Disease, IPF: Idiopathic Pulmonary Fibrosis, KL-6: Krebs von den Lungen-6, MCP-1: Monocyte Chemoattractant Protein-1, NSIP: Non-Specific Interstitial Pneumonia, ROC: Receiver Operating Characteristic, SLX: Carbohydrate antigen Sialyl Lewis (x), SP: Surfactant Protein, UIP: Usual Interstitial Pneumonia

**Table 4:** Studies measuring serum biomarkers in patients with IPF and CVD

**Investigator**	**Patients Controls**	**Biomarker / Summary**	**ROC curve analysis Cut-off values**	**Specificity – Sensitivity Diagnostic accuracy**	**Limitations**
Ohnishi et al. ^51^	33 82	Comparative study of KL-6, SP-A, SP-D, MCP-1 as serum markers for ILDs	Yes KL-6: 465 U/ml Sp-A: 48.2 ng/ml Sp-D: 116 ng/ml MCP-1: 1.080 ng/ml	KL-6: 94 - 96- 96% Sp-A: 86 -82 - 85% Sp-D: 95- 70- 88% MCP-1: 93- 52- 81%	Small number of patients / Non ILD-specific markers / Potential influence by malignancies
Takahashi et al. ^55^	42 108	Serum levels of SP-A and SP-D are useful biomarkers for ILDs in patients with progressive SSc	Yes SP-A: 43.8 ng/ml SP-D: 110 ng/ml	SP-A: 33-100% SP-D: 77- 100%	Small number of patients
Greene et al. ^58^	427 95	Serum SP-A and SP-D as biomarkers in PF of different etiologies	No	Not estimated	Evaluation of serial measurement not definitive
Yanaba et al. ^59^	39	Longitudinal analysis of serum KL-6 levels in patients with SSc: association with the activity of PF	No	Not estimated	Small number of patients / Retrospective study / No ROC curve analysis / cut-off levels
Yanaba et al. ^60^	42	Comparative study of serum SP-D and KL-6 concentrations in patients with SSc as markers for monitoring the activity of PF	No	KL-6: 100 - 39% Sp-D: 88 - 91%	Small number of patients / Retrospective study
Fujita et al. ^62^	37 15	Elevation of CK19 serum levels in patients with IPF associated with CVD	No	Not estimated	Small number of patients / Non ILD-specific marker
Dobashi et al. ^64^	27 10	Elevated serum and BAL CK19 levels in PF and AIP	No	Not estimated	Small number of patients / Discrepancies with other serum parameters
Nakayama et al. ^65^	413 21	CK19 serum levels in patients with nonmalignant respiratory diseases	Yes CK19: 3.5 ng/ml	CK19 : 30 - 50%	Low specificity and sensitivity values / No adjustment with disease severity

Abbreviations: AIP: Acute Interstitial Pneumonia, BAL: Bronchoalveolar lavage, CK19: Cytokeratin fragment 19, CVD: Collagen Vascular Disease, ILD: Interstitial Lung Disease, IPF: Idiopathic Pulmonary Fibrosis, KL-6: Krebs von den Lungen-6, MCP-1: Monocyte Chemoattractant Protein-1, ROC: Receiver Operating Characteristic, SP: Surfactant Protein, SSc: Systemic Sclerosis

Idiopathic pulmonary fibrosis (IPF) is a refractory and lethal ILD characterized by fibroblast proliferation, extracellular matrix (ECM) deposition and progressive lung scarring. The incidence of IPF is estimated at 15–40 cases per 100.000 per year, and the mean survival from the time of diagnosis is 3–5 yr regardless of treatment [47]. On the other hand, scleroderma (progressive systemic sclerosis-SSc), is a systemic disease characterized by a progressive dermatologic abnormality. Systemic involvement may include among others, restrictive lung disease which develops in 30–60% of patients with scleroderma and progresses to severe restrictive lung disease and pulmonary fibrosis (a major cause of death in scleroderma) in 15% of these patients [48]. However, predicting the progression of IPF and SSc as well as their prognosis still remains elusive. To evaluate the activity and monitor the course of the disease HRCT, PFT, BAL and histologic features are clinically used [48]. Nonetheless, there are problems with the sensitivity, effort-dependability and mainly the ease of repetition of these examinations.

One of the pioneering studies that set the foundations for the development of a new research field with massive clinical implications was published ten years ago by Honda et al [49]. Authors were the first reported the potential usefulness of SP-D serum levels in reflecting the disease activity in a group of patients with different types of ILDs including IPF and collagen vascular disease – associated interstitial pneumonia (CVD-IP).

Moreover, KL-6 serological levels have been tested by Kobayashi et al. [50] whether they can reflect the activity of pneumonitis seen in different types of ILD and therefore used as a tool for the differential diagnosis of this large set of diseases and the assessment of their response to treatment. Data derived from this analysis utilizing ROC curves and cut-off values showed a distinct differentiation between ILDs and non-ILDs based on the peripheral KL-6 concentrations as well as a clear correlation of KL-6 levels with the clinical activity of the ILDs as defined by a series of conventional criteria. Although these results create major expectations in the diagnostic field of ILDs, however it should be noted that an elevation of KL-6 and SP-D serum levels suggests diagnosis of ILD and does not establish a specific diagnosis. For these markers to become truly specific, large evaluation and comparative studies are required to determine their usefulness in the differential diagnosis of ILDs.

The first and to best of our knowledge the only so far comparative study testing the sensitivity and specificity of three groups of molecules reported to serve as sensitive markers for ILDs was conducted by Ohnishi et al [51]. They generated ROC curves and performed a comparative analysis of the diagnostic values of KL-6, SP-A, SP-D and MCP-1 in a pool of serum samples derived from patients with IPF and CVD-IP. Intriguingly, this study demonstrated for the first time a clear superiority of KL-6 as a diagnostic marker of ILDs in terms of diagnostic accuracy, sensitivity, specificity and likelihood ratio. Interestingly, results from the statistical analysis confirmed that all four serum markers are specific markers for IPF and at least superior to previously described markers such as lactate dehydrogenase and other collagen products [52]. Although this study adds knowledge of high scientific rigidity about the status of these peripheral markers as diagnostic tools, however there are substantial weaknesses arising from the origin disadvantages of these molecules as specific biologic markers. More specifically, serum levels of all the four markers can be influenced by the presence of ILDs other than IPF as well as lung disorders other than ILDs such as malignancies, systemic inflammations and fibrosing lung infections [12,53,54]. Nonetheless, these observations are not to diminish their value as diagnostic tools but to illuminate the need for their further investigation in the context of more detailed studies searching potential correlation of these markers with the clinical and radiological findings and adding more specific information on the differential diagnosis of IPF among the other 200 members of the ILD group.

Towards this direction Takahashi et al. [55,56] were the first who attempted to prove a correlation between the SP-A and SP-D circulating concentrations and the radiologic findings as well as the disease extent in patients with CVD-IP and IPF. Authors published two really well done and heavily informative papers, both of them are reviewed here.

In the first study [55], results derived by a statistical analysis using ROC curves and cut-off levels demonstrated that elevated levels of SP-A and SP-D in patients with progressive SSc closely reflect in terms of sensitivity and specificity the presence of ILD on the basis of CT diagnosis. Moreover these markers increase even in patients with mild alveolitis not detectable by compatible chest X-ray. Additionally an interesting finding with major clinical applications pointed out by authors is that the combination assay of SP-D circulating concentrations and chest X-ray is almost equivalent to CT in the detection of ILD and can potentially serve as a contributor to reduce the risk of clinicians overlooking ILD complicated by progressive SSc.

Furthermore, Takahashi et al. [56] estimated the serological levels of SP-A and SP-D in IPF patients and correlated them with the radiological and functional parameters. Interestingly, accumulated findings from this analysis demonstrated a positive correlation between serum levels of both markers and the extent of alveolitis whereas no correlation was observed with the progression of fibrosis. Moreover authors showed a statistically significant link between increased concentrations of SP-D and rapid decline in pulmonary function tests as well as with higher rates of mortality. The above findings suggest that these proteins and especially SP-D may help scientists to shed further light in the pathologic characteristics of IPF seen in HRCT and assist as prognostic tool for the final outcome in applying optimal therapeutic approaches. However, caveats that should be taken under consideration include weak correlations of SP-A and SP-D serum levels with the CT features and inconclusive analysis of the disease mortality and the biomarker serum levels.

Several biologic markers have been studied the past few years not only to evaluate the disease activity and for differential diagnosis of ILDs but also for prediction of the disease outcome and the effectiveness of the commonly applied treatment during the accelerated phase of pulmonary fibrosis.

One of the most detailed and informative study in this field was published by Yokoyama et al [57]. Scrutinizing for early predictive markers of the therapeutic effects of high-dose corticosteroids on patients with rapidly deteriorating IPF, authors demonstrated that circulating levels of KL-6 could predict the efficacy of corticosteroids at an earlier time-point than other studied non-specific markers, when overall clinical effect may not yet be evident. However, potential criticisms of this study including the small number of a specific group of IPF patients studied, the finding that the KL-6 levels in the pretreatment period could not be used to predict outcome as well as the evidence that KL-6 concentrations are affected by certain malignancies [12,53] pose major limitations to these observations and highlight the necessity for further investigation and studies.

Currently, the hurdle faced by young physicians that limits the application of blood biomarkers in the daily clinical practice of IPF patients is whether alterations of their circulating concentrations are specific for IPF or they also reflect other lung parenchyma abnormalities seen in different ILDs. Several studies have tested the efficacy of blood biomarkers in differentiating IPF from other ILDs. Some of them are reviewed here.

One of the first and most extensive studies addressing that important issue was conducted by Greene et al. [58] in the context of a large cohort multivariate analysis including over 200 patients with IPF and progressive SSc and approximately 200 patients with other ILDs. Notably, authors showed an important correlation between elevated SP-A and SP-D serological levels in IPF and SSc patients compared to patients with sarcoidosis or beryllium disease. Furthermore, SP-D levels found to be strongly related to radiographic abnormalities in patients with IPF. The most intriguing result of this study was that both increasing SP-A and SP-D levels were highly predictive of mortality in patients with IPF and progressive SSc and this finding robust (especially SP-D levels) after adjustment for many measures of disease severity. However, a major limitation of this analysis was that serial measurement of SP-A and SP-D levels during the clinical course of patients with diffuse lung disease was not definitively evaluated. Hence, an important issue highlighted by authors that needs further investigation is the usefulness of serum alterations in reflecting the disease activity and extent during different time-points of the disease course.

To this end Yanaba et al. [59] carried out a longitudinal retrospective study in a relatively small number of subjects with SSc and found that the majority of patients with normal baseline serum KL-6 levels exhibited no deterioration or new onset of PF whereas patients with dramatically increased KL-6 levels showed a parallel progression of PF.

The latter observation concerning the utility of KL-6 as a biomarker in monitoring the clinical course of PF was further strengthened by the same group of scientists {Yanaba et al. [60]} in the context of a comparative study of SP-D and KL-6 serum levels in a limited number of patients with SSc. Moreover, by generating ROC curve analysis, authors concluded that the combined use of these two blood markers could be of higher diagnostic and monitoring value for the activity of PF than the single use of each marker. However, data derived from the last two studies was inconclusive mainly due to the fact that it was produced by retrospective analyses and the number of patients was inadequately small for any meaningful outcome.

Alternatively, Ishii et al. [61] estimated the diagnostic values of five lung peripheral biomarkers including SP-A, SP-D, KL-6 and two tumour markers in discriminating patients with ILD of various histopathologic patterns such as usual interstitial pneumonia (UIP) and non-specific interstitial pneumonia (NSIP). Ultimately authors demonstrated differential SP-A serum concentrations between patients with UIP and NSIP indicating a potential role of this protein for the discrimination of these two members of ILD group with sometimes similar pathologic and radiologic characteristics but with totally different prognosis and response to treatment. Although this study adds critical information on the field of differential diagnosis of ILDs, however it is of high risk to adapt this evidence in the everyday clinical practice and make the differential diagnosis based only on non-invasive methods. It is of high importance to note that there are substantial weaknesses including the relatively small number of patients used and the overlap in the serum SP-A levels between UIP and NSIP patients that limit the further application of these results and illuminate the need for a more extensive analysis to determine its diagnostic cut-off levels.

To gain a more comprehensive understanding on the activity of lung epithelial cell damage and repair characterizing IPF and other ILDs and to clear out whether these are related to the disease prognosis, investigators studied the significance of other lung specific epithelial proteins initially reported as tumour markers, in reflecting these pathological abnormalities. Some of the most extensively reported markers were CK19 and carbohydrate antigens sialyl Lewis (Ca 19-9, SLX).

There have been several published papers reporting elevated levels of CK19 in the serum of patients with various types of ILD [62,63]. Nevertheless, the first report in the literature demonstrating elevated CK19 serum levels in patients with different types of ILDs and a significant association with several clinical parameters known to be strong predictors of the disease clinical course was made by Dobashi et al [64]. This data combined with results from immunohistochemical analysis indicate that CK19 is a marker of lung injury. However, it is inconclusive given the small sample of patients recruited and the major discrepancies between levels of CK19 and other serological predictive parameters. Therefore, the efficacy of CK19 in the differential diagnosis of IPF from other ILDs as well as in the prediction of the disease prognosis should be further evaluated.

Fueled by this prospect, Nakayama et al. [65] were the first that revealed a positive linkage between increased CK19 serum levels and low survival rate in patients with IPF and CVD-IP suggesting a potential role for this tumour marker in reflecting the severity of the lung injury and the disease prognosis. Additionally, this study clearly documented by using ROC curves and cut-off levels the ineffectiveness of this marker to differentiate IPF from other non-malignant respiratory diseases characterized by marked epithelial cell damage. This observation coupled with substantial weaknesses including the lack of adjustment of CK19 serum levels with the disease severity, allow us to make only speculations on the exact role of this biomarker as a prognostic factor and warrant consideration.

Elevated plasma levels of Ca 19-9 were first demonstrated by Bungo et al. [66] in 1988. In addition, Mukae et al. [67] reported two cases of interstitial pneumonitis with marked increase of carbohydrate antigens in serum and BALF, the level of which changed in accordance to their clinical course. Immunohistochemical analysis showed the expression of these antigens on bronchiolar epithelial cells and regenerating epithelial cells which covered the surface of fibrosing alveolar septi or remodeling septal structures finding that was further corroborated by Shimizu et al. [68] who stated elevated serum levels of cancer-associated antigens (Ca 19-9, SLX) primarily localized in the hyperplastic bronchiolar epithelium and the surface epithelium cells of microscopic honeycombing in two different patients with IPF. However, the first study that tested the clinical usefulness of elevated blood levels of Ca 19-9 in a series of patients with fibrosing lung disease was conducted by Satoh et al [69]. In consistency with previous reports in the literature {Takayama et al. [70]}, authors demonstrated a strong correlation of serum levels with the degree of disease activity indicating a potential role of these molecules as prognostic markers of disease severity and therapeutic response. Furthermore, Yokoyama et al. [71] carried out the first comparative evaluation of the diagnostic values of three serum carbohydrate antigens (KL-6, Ca 19-9, and SLX). They plotted ROC curves and demonstrated a clear superiority in terms of specificity, sensitivity and diagnostic accuracy of KL-6 plasma levels in discriminating interstitial pneumonia from alveolar pneumonia and healthy volunteers. In another study, Satoh et al. [72] coupled SLX serum cut-off levels with Western blotting analysis and documented that this combination is of high diagnostic power in differentiating IPF from lung adenocarcinoma.

Evidence presented give credence to the view that cancer-associated antigens could serve as valuable and reliable diagnostic and prognostic markers of ILDs. On the other hand, none of the studies provided us with established diagnostic cut-off values since they exhibited low statistical power and in most of them no adjustment with the disease severity was performed. Moreover, KL-6 has proven to have higher discriminative power than carbohydrate antigens. In addition, potential arguments include the lack of knowledge regarding the role of these markers in the pathogenetic pathway of IPF. So far, the only study addressed this crucial issue was published by Obayashi et al. [73] who reported a direct correlation of elevated BALF Ca 19-9 levels with the percentage of neutrophils suggesting a potential role of carbohydrate antigens as neutrophilic chemoattractants and subsequently as contributors in the process of lung injury. Further analyses in the context of large prospective studies are warranted to elucidate this role and establish their clinical usefulness as markers of disease severity and activity.

The past few years led by the same perspective idea to find a reliable and reproducible biologic marker for the diagnosis and prediction of ILDs, several group of scientists have tested the usefulness of a slew of cytokines to play that role.

One of these reports was published by Suga et al [74]. Authors concluded that elevated serum and BAL levels of MCP-1 could be useful to discriminate IPF among other members of the ILD group. Interestingly, in circulating MCP-1 concentrations of patients with IPF underwent steroid therapy no trend towards fall with treatment was noticed whereas a major fluctuation in pre-and post-treatment levels was readily identifiable. However, these results do not prove neither the reproducibility of this biormarker nor its relationship with the disease behaviour. The latter observations coupled with the evidence that MCP-1 levels are markedly influenced by corticosteroid treatment as well as other lung disorders since it is a mediator of inflammation also produced in other parts of body, limit its specificity in the differential diagnosis of ILDs and underline the need for re-evaluation of its predictive value.

One of the most exciting roles cytokines can play is their use as indicators of the therapeutic effects of several drugs against IPF providing investigators with useful knowledge on the potential mechanisms of these drugs in terms of physiology and molecular biology highlighting novel therapeutic targets.

The first study in humans to characterize the effects of IFN-γ1b on plasma and lung biomarkers, speculated to serve as critical mediators in the pathogenesis of IPF was recently published by Strieter et al [75]. Authors documented indefinite pre- and post-treatment alterations in the peripheral concentrations of several biologic markers associated with fibrosis, aberrant vascular modelling and immunomodulatory activity that can be used only for generating hypotheses for future research. However, the strongest finding in this study was the differential elevation of blood and BAL before and after treatment levels of ITAC/CXCL-11, a chemokine with multifunctional activities including antiangiogenic and antimicrobial. Inferentially, this study comprises evidence that mortality in patients with IPF could be potentially improved through the versatile protective properties of IFN-γ1b supporting its therapeutic utility (Tables 3 and 4).

## 2. Serum biomarkers in occupational and environmental diseases (Table 5)

**Table 5:** Studies measuring serum biomarkers in patients with occupational and environmental diseases

**Investigator**	**Patients Controls**	**Biomarker / Summary**	**ROC curve analysis Cut-off values**	**Specificity – Sensitivity Diagnostic accuracy**	**Limitations**
Borm et al. ^80^	33 58	Glutathione levels reflect early inflammatory response but are not a predictive parameter for individual susceptibility in CWP	No	Not estimated	Retrospective analysis / No serial measurement / Small number of patients / Scarce data on the interrelationships among antioxidant enzymes
Borm et al. ^81^	39 27	TNF is a marker of individual susceptibility to dust-induced lung fibrosis in coal-workers	No	Not estimated	Retrospective analysis / Small number of patients / Indefinite data on the acquired or genetically controlled differences of TNF release
Schins et al. ^83^	104 29	A 5-year follow-up study reveals that TNF is a reliable prognosticator of CWP	No	Not estimated	No linkage to disease behaviour / Indefinite data on the effect of exposure / No ROC curve analysis / cut-off levels
Schins et al. ^84^	104 29	Serum PIIIP has a poor value as a biomarker to screen for CWP	No	Not estimated	Studied population: retired miners / Diverse exposure to coal-dust / Heterogeneity of disease-fibrotic activity / Indefinite data on the effect of exposure and time variation in serum PIIP
Cobben et al. ^86^	201 48	Serum LDH levels are elevated in coalminers but are not associated with clinical variables of disease severity	No	Not estimated	Retrospective study / No serial measurements / No ROC curve analysis / cut-off levels
Cobben et al. ^87^	191 48	Serum b-glucuronidase levels may be a useful biomarker in monitoring lung inflammation following coal dust exposure	No	Not estimated	Retrospective study / No serial measurements / No ROC curve analysis / cut-off levels
Takahashi et al. ^88^	35 237	Elevated serum KL-6 levels in patients with FLD	Yes KL-6: 410 - 450 U/ml	KL-6: 80 - 73%	Small number of patients / No serial measurements
Harris et al. ^89^	108 20	Elevated serum neopterin levels in CBD	Yes Neopterin: 1.27 ng/ml	Neopterin: 88 - 58%	No serial measurements / No correlation with clinical and/or radiological parameters
Maier et al. ^90^	31 34	Neopterin is a useful diagnostic tool in differentiating CBD from BeS	Yes Neopterin: 2.5 ng/ml	Neopterin: 100 - 80%	No serial measurements Poor prognostic value Indefinite cut-off levels

Abbreviations: BeS: Beryllium Sensitization, CBD: Chronic Beryllium Disease, CWP: Coal-Workers Pneumoconiosis, FLD: Farmer's Lung Disease, KL-6: Krebs von den Lungen-6, LDH: Lactate Dehydrogenase, PIIIP: type III procollagen peptide, ROC: Receiver Operating Characteristic, TNF: Tumor Necrosis Factor

The concept of biomarkers is extensively developed in the field of occupational and environmental medicine. Historically, in epidemiologic research, the link between exposure and disease was often without knowing the mechanism or intervening events. The past few years, the identification of lower levels of exposures and the effective clinical and public health management of high risk populations has drugged much of attention. Borm [76] highlighted the need for methods to monitor early adverse effects, exposure, and/or susceptibility of individual subjects due to occupational and environmental causes and Schulte and Perera [77] nicely reviewed the need of extending the use of biomarkers to population studies. The relative inability of the current modalities including PFTs, questionnaires and physical examinations, to detect early signs of occupationally and environmentally related adverse effects has prompted much interest in using biochemical, molecular and pathologic changes as indicators for respiratory diseases [78]. The conventional approach to validate a biomarker is to relate a critical effect to exposure or calculated dose. A positive outcome judged on the marker-effect relationship leads to nomination of the event as a biomarker. An essential requirement for a successful biomarker of effect or susceptibility is that it should identify from among all exposed individuals those most likely to become diseased. Fueled by this prospect, a framework of studies has been designed to utilize biomarkers of exposure, susceptibility and pathophysiological changes as part of the array of tools available to assess environmental disease.

Borm and co-workers [79,80] evaluated the clinical usefulness of antioxidant enzymes, (TNF) and serum type III procollagen peptide in studies of coal dust-induced lung disorders, a wide spectrum of diseases including chronic inflammation and progressive massive fibrosis (PMF). In the first case-control study [79] glutathione levels were decreased in early stage coal workers pneumoconiosis (CWP) but increased in patients with PMF indicating a potential role of anti-oxidant enzymes in the early detection of inflammatory response to mineral dust exposure. However, serum alterations of glutathione failed to predict individual susceptibility since they most likely reflect a consequence of the disease. With this aim in mind, authors carried out a second case-control study [80] where TNF was studied as a potential risk factor and concluded that TNF release from peripheral blood monocytes exposed coal mine dust was a marker of individual susceptibility to dust-induced lung fibrosis. The latter observation corroborated earlier findings [81,82] and supported the notion that TNF release from monocytes or TNF in plasma are not associated with actual or cumulative exposure. However, definitive proof must come from a prospective analysis in the context of carefully designed follow-up study. The only follow-up study that has been conducted so far {Schins et al. [83]} showed that the miners that had disease progression (PMF) during 5 years already had high levels of dust-induced monocyte TNF release at the beginning and no alterations during follow-up were noticed. This highly informative study excluded TNF as an exposure marker and suggested that TNF release is a constitutional marker of the disease prognosis and is not highly affected by the disease itself. In a fourth study, Schins and Borm [84] conducted a 5-year prospective analysis and estimated whether type III procollagen peptide could serve as a valuable predictor of the fibrotic lung disease progression, outcome or activity. Since no differences between procollagen serum levels in coal-miners and non-dust-exposed controls were observed, the investigators stated that type III procollagen peptide is not a reliable marker of early effect.

To characterize the nature and extent of coal dust induced airway injury there is a need for biomarkers useful to monitor exposure effects. The potential of many cell mediators as monitoring tools i.e. CC16 [85], antioxidants [79] and several cytokines [80] has been raised many times. Towards this direction Cobben et al. [86] studied the role of LDH (a marker of cell damage) as marker of lung tissue injury. Authors conducted the first human study describing a considerable elevation of this enzyme in a group of ex-coalminers and a further association with other clinical variables. Additionally, the same group of investigators [87] evaluated the role of b-glucuronidase as marker of phagocytic activity and reported increased plasma concentrations in a group of coal-miners after 20 years of exposure whereas no correlation with clinical parameters was observed. The latter data is in line with the hypothesis that LDH and b-glucuronidase activity are conceivable markers of disease activity. Potential criticisms include the retrospective analysis, the lack of serial measurement and link to disease behaviour. Thus, these results should be interpreted with care. To determine the significance of these biological indicators with regard to the development or progression of CWP a longitudinal prospective design is necessary.

Hypersensitivity pneumonitis also called extrinsic allergic alveolitis is an ILD that may be due to a wide variety of inhalative antigenic stimuli. To date, the conventional diagnosis of hypersensitivity pneumonitis is usually made on the basis of history of periodically recurring or permanent complaints upon exposure to a specific inhalative antigen, interstitial abnormalities in both lungs by chest radiography or HRCT and detection of precipitating antibodies. However, at present, there are few diagnostic procedures available to confirm the diagnosis and to estimate the disease activity and most of them are too invasive and costly for widespread and daily use. With this aim in mind, Takahashi et al. [88] evaluated serum KL-6 measurement as a biologic marker for farmer's lung disease (FLD), a type of hypersensitivity pneumonitis caused by the inhalation of moldy antigens. Authors conducted a large cohort study and clearly documented significant higher blood KL-6 levels in patients with FLD compared to the levels of farmers with or without precipitating antibodies. In addition, major findings in this study previously unknown, include the consistency between KL-6 serum levels in FLD patients and the activity of the disease as well as the indication that elevated KL-6 concentrations coupled with conventional diagnostic criteria may detect subclinical FLD and determine early and effective therapeutic interventions. However, to validate whether serum KL-6 levels reflect the disease activity a larger evaluation of the specificity and sensitivity of this marker against the disease behaviour in combination with a definitively estimated serial measurement of its plasma levels are required.

While its direct role in pathogenesis of immune and inflammatory responses remains unclear, neopterin's ability to reflect monocyte and macrophage activation has been exploited and yet postulated as a marker of the beryllium-specific cell-mediated immune response that leads to a chronic granulomatous disease, a hypersensitivity disorder named chronic beryllium disease (CBD). In the study of Harris et al. [89] ROC curves were generated to evaluate the optimum diagnostic accuracy of neopterin's cut-off levels. Interestingly, authors have found that the combination of elevated neopterin's serum levels with the conventional screening test for CBD, beryllium lymphocyte proliferation test exhibited an optimized positive predictive value suggesting neopterin as a valuable biomarker in discriminating workers with CBD from these that are only sensitized to beryllium rendering lung biopsy unnecessary. These results were substantiated by in-vitro studies of peripheral blood mononuclear cells derived by patients with CBD or beryllium exposed workers [90] and an association between serum levels of neopterin and clinicolaboratory parameters of the disease severity was found. However, further confirmatory tests in large cohorts of patients including serial measurements and correlation of results with clinical and radiological findings are essential to determine reliable cut-off values for the diagnosis of the disease and the assessment of the progression likelihood (Table 5).

## 3. Serum biomarkers in other interstitial lung diseases (Table 6)

**Table 6:** Studies measuring serum biomarkers in patients with other ILDs

**Investigator**	**Patients Controls**	**Biomarker / Summary**	**ROC curve analysis Cut-off values**	**Specificity – Sensitivity Diagnostic accuracy**	**Limitations**
Ohnishi et al. ^92^	30	Elevated circulating KL-6 levels in patients with drug induced pneumonitis	No KL-6: 520 U/ml	Sensitivity: 53 – 89%	Small number of patients / Discrepancies with CT features
Kohno et al. ^93^	15	Circulating antigen KL-6 and LDH for monitoring irradiated patients with lung cancer	No	Not estimated	Small number of patients / Retrospective study / Evaluation of serial measurement not definitive
Goto et al. ^94^	16	Serum levels of KL-6 are useful biomarkers for severe radiation pneumonitis	No	Not estimated	Small number of patients / Retrospective study / Chemotherapy influence
Takahashi et al.^95^	25	Diagnostic significance of SP-A and SP-D in sera from patients with radiation pneumonitis	No	Sp-A: 83 - 85% Sp-D: 83 - 85%	Small number of patients / Chemotherapy influence / No long term follow-up / Non ILD-specific markers
Al-Salmi et al. ^97^	10 10	Elevated serum KL-6 and SP-A and SP-D in pediatric ILDs	No	Not estimated	Small number of patients / Diversity of the diseases studied / Poor correlation with functional and radiological parameters

Abbreviations: BAL: Bronchoalveolar lavage, CBD: Chronic Beryllium Disease CT: Computed Tomography, FLD: Farmer's Lung Disease, ILDs: Interstitial Lung Diseases IPF: Idiopathic Pulmonary Fibrosis, KL-6: Krebs von den Lungen-6, LDH: Lactate Dehydrogenase, ROC: Receiver Operating Characteristic, SP: Surfactant Protein

Since lung specific-epithelium proteins reflect the epithelial damage and turnover it has been hypothesized and ultimately demonstrated that they can be used as effective circulating markers for the diagnosis and prognosis of the clinical course of various types of interstitial pneumonitis including drug-associated, radiation-induced and hypersensitivity pneumonitis. Furthermore, pneumoproteins have already been introduced as potential valuable biomarkers in the research field of pediatric ILDs.

Numerous agents including cytotoxic and non-cytotoxic drugs exert pulmonary toxicity including interstitial pneumonitis which several times culminates to a fatal outcome [91]. Therefore early diagnosis is crucial, since withdrawal of the implicated drug is usually the most sufficient treatment for drug toxicities, whereas undiagnosed toxicity can be progressive and fatal.

Ohnishi et al. [92] in their attempt to introduce a specific and reproducible hallmark for the recognition of different types of drug-induced lung injury and the prediction of their clinical course, estimated the plasma concentrations of KL-6 in 30 patients with drug-associated pneumonitis classified into four different predominant radiographic patterns. The remarkable ascertainment of this study was the demonstration of a high sensitivity relation between elevated serum KL-6 levels and particular types of lung injury as well as with their clinical course. However, the small number of patients included in this study coupled with discrepancies between serum KL-6 levels and the disease extent as defined by CT findings comprise major caveats and illuminate the need for further prospective studies to determine whether measurement of KL-6 levels would be beneficial in the monitoring and early detection of drug-induced ILDs.

Radiation pneumonitis is the most common complication for thoracic tumours and is classified as an ILD. Since CT scanning that represents the gold standard diagnostic procedure for radiation pneumonitis is either not frequently repeatable or often exhibits non-specific findings hardly to be differentiated from the lung tumour manifestations, a lung specific laboratory test easily repeatable and reproducible is highly required for the early detection of radiation-associated lung injury. On the basis of this conception, Kohno et al. [93] were the first reported that serum KL-6 levels are a sensitive marker for detecting severe radiation pneumonitis. Nevertheless, because authors measured KL-6 serum levels at a few time-points in each patient's clinical course, data derived from this study is inconclusive and cannot be applied to firmly correlate circulating KL-6 concentrations with the clinical course of radiation pneumonitis. Therefore, Goto et al. [94] to further streamline these observations retrospectively monitored at shorter intervals blood KL-6 levels in patients with lung cancer who had received radiotherapy with or without chemotherapy and showed a correlation with the clinical course of radiation pneumonitis and the response to treatment. Interestingly, the finding that serum KL-6 levels in some patients were increased priory to the clinical and radiological diagnosis of radiation pneumonitis is particularly noteworthy and should be kept in mind.

Another study searching for potential indicators for the early detection of radiation pneumonitis and for monitoring its clinical course was conducted by Takahashi et al [95]. Remarkably almost all of the patients with radiation pneumonitis detected by HRCT exhibited significant increases in both SP-A and SP-D levels which showed high sensitivity and specificity for the early diagnosis of radiation pneumonitis compared with other conventional haematological laboratory indices. Additionally, an agreement of SP-A and SP-D plasma levels with the clinical response to steroid therapy was also noted. This data suggest a possible role of lung-specific epithelial proteins as a diagnostic tool for the recognition of radiation-induced lung injury even when its radiographic change is faint.

However, these studies exhibit major caveats that must be addressed prior to their further application in the routine clinical practice. Briefly, we report the small number of patients studied, the application of chemotherapy prior to radiotherapy, the absence of serial measurements through the clinical course of the disease and the evidence that these proteins (SP-A and KL-6) are neither organ nor disease specific since they have also been used as markers for lung adenocarcinoma [18,53]. To confirm the assumptions arising from these concerns, further studies are required.

As mentioned above the spectrum of ILDs includes a large heterogeneous group of disorders calculating over 200 members with IPF to be the most common form of idiopathic ILD. In contrast ILD in children occurs far less frequently and there are no dominant forms [96]. Although this research field is newly developed a recently published study by Al-Salmi et al. [97] revealed for the first time elevated serum levels of three candidate biomarkers of the disease activity and severity including KL-6, SP-A and SP-D in a group of children with ILDs of various histopathologic patterns. These results corroborate earlier findings by Kobayashi et al. [98] who reported elevated KL-6 serum levels in three children with ILD associated with dermatomyositis. Nonetheless it should be noted that these studies are deficient because of the small number of patients recruited and the diversity of the diseases studied. Furthermore, data described here is inconclusive and it ascertains poor correlation with the functional and the radiological parameters. More in depth analysis in a large cohort of patients is required for any meaningful outcome (Table 6).

## 4. Serum biomarkers in sarcoidosis (Table 7)

**Table 7:** Studies measuring serum biomarkers in patients with sarcoidosis

**Investigator**	**Patients Controls**	**Biomarker / Summary**	**ROC curve analysis Cut-off values**	**Specificity / Sensitivity Diagnostic accuracy**	**Limitations**
Ziegenhagen et al.^104^	77 50	TNF-a release from alveolar macrophages and serum level of sIL-2R are prognostic markers for sarcoidosis patients	No	Not estimated	No serial measurements / No ROC curve analysis / cut-off values
Ziegenhagen et al. ^105^	73 48	BAL and serological parameters reflect the severity of sarcoidosis	No	Not estimated High predictive value for neopterin+ sIL-2R	No serial measurements / No ROC curve analysis / cut-off values
Grutters et al. ^106^	47	Positive correlation between sIL-2R serum levels and the disease activity and severity in patients with sarcoidosis	No	Not estimated	Small number of patients with serial measurement / No ROC curve analysis / cut-off values
Rothkrantz-Kos et al.^109^	144 282	Potential usefulness of inflammatory markers to monitor respiratory functional impairment in sarcoidosis	Yes sIL-2R: 750 U/ ml sIL-2R: 1300 U/ml	sIL-2R: 94 - 82% sIL-2R: 82 - 94%	Discrepancies between treated and untreated patients / No serial measurements / Retrospective study / No correlation with radiological findings
Hashimoto et al. ^117^	26	Correlation of plasma MCP-1 and MIP-1a levels with disease activity and clinical course of sarcoidosis patients	No	Not estimated	Small number of patients / No ROC curve analysis / cut-off values / No determination of the cytokines' cellular sources
Iyonaga et al. ^118^	47 10	MIP-1 serum levels estimate the activity of granuloma formation in sarcoidosis	No	Not estimated	Small number of patients / No ROC curve analysis / cut-off values / No correlation with radiological findings
Kobayashi et al. ^119^	47	Serum KL-6 for the evaluation of active pneumonitis in pulmonary sarcoidosis	No	Not estimated	Small number of patients / No follow-up laboratory data
Hermans et al. ^120^	117 117	Serum CC16 levels, a marker of the integrity of the air-blood barrier in sarcoidosis	No	Not estimated	Non ILD- specific marker / Potential influence by tobacco smoking / Poor discriminative value
Janssen et al. ^122^	79 38	Elevated serum CC16, KL-6, and SP-D levels reflect pulmonary disease severity and prognosis in sarcoidosis patients	Yes CC16: 12.7 ng/ml KL-6: 223 U/ml SP-D: 91.7 ng/ml	CC16: 84 - 73- 73% KL-6: 84 - 86 - 86% SP-D: 84 - 66 - 66%	Non ILD- specific markers / Retrospective study / No serial measurement

Abbreviations: CC16: Clara Cell protein 16, ILD: Interstitial Lung Disease, KL-6: Krebs von den Lungen-6, MCP-1: Monocyte Chemoattractant Protein-1 MIP-1a: Monocyte Inflammatory Protein-1a, ROC: Receiver Operating Characteristic, sIL-2R: soluble Interleukin-2 Receptor, SP: Surfactant Protein, TNF-a: Tumour Necrosis Factor-alpha

Sarcoidosis is a chronic systemic disorder characterized by the presence of non-caseating granulomas and accumulation of T-lymphocytes and macrophages in multiple organs [99]. The mechanisms leading to the persistent accumulation of inflammatory cells and maintain the alveolitis which may lead to irreversible organ damage are not fully understood. The classical parameters used in the management of pulmonary involvement are mainly radiological methods and PFTs which do not gauge alveolitis but rather pulmonary impairment. Consequently these parameters have been proved ineffective both for early diagnosis of the disease and prediction of the response to treatment. The last twenty years immunological studies performed with cells obtained by BAL have shed further light into the pathogenetic mechanisms of sarcoidosis [100,101] and formed the basis of concepts of its immunopathogenesis. To further streamline these concepts and ameliorate hardships generating from their clinical application, several serological parameters including cytokines, soluble cytokine receptors, enzymes and other serum components including lung epithelium-specific proteins were delineated to probe different aspects of inflammatory activity and therefore reflect the disease severity and help the early diagnosis of progressive disease [45]. Although levels of these markers are closely associated with the pathogenesis of the disease [102,103], however the availability of sufficient information as to which of them is valuable for assessment of the disease severity and the prediction of clinical deterioration still represents a bottleneck. From a clinical point of view it is even more important to know whether sarcoidosis is severe, rather than active. The latter observation represents the key evidence determining the initiation of treatment. Some of the most extensive and informative studies addressing this crucial issue are presented and criticized in this review article.

One of the first studies attempted to circumvent this problem was published by Ziegenhagen et al [104]. Authors scrutinized the efficacy of both BAL and serum parameters in indicating likelihood of progression in patients with sarcoidosis and revealed that almost half of the patients with no indications for steroid therapy who had elevated sIL-2R plasma levels experienced disease deterioration whereas none with normal values did. The aforementioned observation strongly suggests that this immune parameter could serve as a prognostic guide leading to the identification of patients with greater risk to relapse and maybe associated with clinical findings such as the disease course.

The effectiveness of sIL-2R in evaluating sarcoidosis severity even in the early stages of the disease was further confirmed by another study of the same group of scientists [105] who clearly reported significantly elevated sIL-2R concentrations in patients with progressive sarcoidosis. On the contrary, this evidence was followed by a surprising finding regarding the ACE serum concentrations which did not differ significantly between patients with stable or progressing disease indicating a poor predictive value of this biomarker in sarcoidosis.

In the study of Grutters et al. [106] authors evaluated the serological concentrations of sIL-2R as marker of disease activity, severity and prognosis in a well-defined group of patients with sarcoidosis. Data derived from this study partially consistent with other studies [107] asserts a positive correlation between sIL-2R serum levels and the disease activity and severity and furthermore claims a predictive value for this serum biomarker. However, this study was retrospective and major discrepancies between the latter findings and results derived from older studies [108] were also notable.

To establish a more reliable association between sIL-2R and severity of sarcoidosis Rothkrantz-Kos et al. [109] applied ROC curve analysis and estimated the potential prognostic and diagnostic value of four inflammatory markers to predict respiratory severity in a large number of sarcoidosis patients. Areas under curve indicated that sIL-2R showed the greatest ability to determine pulmonary severity as assessed by the highest sensitivity, specificity, positive and negative predictive values among the evaluated markers, including C-reactive protein, serum amyloid A and ACE. Nonetheless, discrepancies between serum levels of all markers in treated and untreated patients coupled with the lack of serial measurement through patients' clinical course pose major limitations to the reported data which is unable to determine the potential efficacy of the evaluated markers to reflect respiratory severity in sarcoidosis patients under treatment in general.

Accumulated evidence from the data presented reveal that the predictive values of serum ACE in sarcoid patients are still under consideration and findings derived from these studies are controversial concerning its usefulness in assessing the disease severity and predicting the clinical outcome. Although some investigators suggest that a rising ACE serum level can predict radiographic relapses of sarcoidosis [110] others have clearly demonstrated that ACE appears to be of poor prognostic value [111,112]. In the aforementioned studies [104,105,109] ACE does not yield prognostic information. However, several studies in the literature have addressed this issue and have reported an extraordinary high variability in serum ACE concentration in health, which is provoked by numerous factors. The most important factor, responsible for approximately 25% of this variation is a deletion/insertion polymorphism in intron 16 of the ACE gene [113-116]. The question whether genotype corrected normal values might be able to increase the reliability of ACE serological concentrations as a marker of disease activity in sarcoidosis remains to be determined by further studies.

The role of chemokines in monitoring the disease behaviour and in reflecting the activity of granuloma formation in sarcoidosis patients has been evaluated and postulated. Hashimoto et al. [117] were the first who carried out a longitudinal evaluation of plasma MCP-1 and MIP-1a levels in a rather small number of sarcoid subjects and demonstrated a closely relation between serum levels of the studied biomarkers and the clinical course of the disease as defined by radiological and laboratory parameters. However, further light should be shed onto the role of these chemokines as markers of the disease activity and severity as well as their cellular sources and their relationship with the granuloma formation.

To this end Iyonaga et al. [118] in parallel with the elevation of MCP-1 serum levels in sarcoid individuals and their linkage with radiographic abnormalities showed by both immunohistochemistry and in situ hybridization that the origin of the increased MCP-1 plasma levels were the macrophages peripheral to the granulomas suggesting a role for this molecule as an indicator in estimating the activity of granuloma formation and subsequently of the sarcoidosis activity.

Apart from their application as prognostic and diagnostic tools of IPF and other ILDs the utility of lung-epithelium specific proteins including KL-6, SP-D and CC16 as biological markers of the disease activity has been separately evaluated in sarcoidosis patients. To date, several studies have addressed this issue and much good work has been done towards this direction.

The potential usefulness of pneumoproteins and more specifically KL-6 as true marker in the assessment of sarcoidosis was initially suggested by Kobayashi et al [119]. Authors were the first demonstrating linkage between increased KL-6 plasma levels and consistent changes in conventional clinical and laboratory parameters for the evaluation of alveolitis activity in sarcoidosis patients suggesting a potential role of KL-6 as an indicator of the disease severity.

Moreover Hermans et al. [120] scrutinized the role of CC16 as a valuable tool for the non-invasive evaluation of the damage and consequently the integrity of the air-blood barrier associated with sarcoidosis, shedding further light into pathogenetic mechanisms underlying the disease severity. Among other findings authors demonstrated that CC16 serum levels are influenced by the pulmonary extent of the disease as defined by the radiological abnormalities indicating a potential role of this molecule as a non-invasive and easily reproducible parameter for the assessment of the disease severity. On the other hand, blood levels of this protein reflect not only the rate of entry into the circulation, but also the rate of clearance since it has been reported that CC16 is rapidly eliminated by glomerular filtration [121]. Authors addressed this issue and found that the increase occurred independently of the impaired renal function, attributing the elevation to the leakage of the protein into the bloodstream across the air-blood barrier. Further studies are warranted to establish this notion.

Although these markers have been studied separately [41,119,120] no study so far had performed a comparative analysis of their diagnostic and prognostic accuracy. Triggered by the latter perspective idea Janssen et al. [122] recently published a really well done and informative paper comparing the ability of these three markers to reflect pulmonary disease severity and prognosis in sarcoidosis patients. They performed a ROC curve analysis which revealed a better sensitivity for KL-6 in discriminating sarcoidosis patients, results consistent with previous findings [51]. However, as it pointed out from the authors KL-6 and CC16 are not ILD-specific markers [[26,53], and [121]] and evidence that supports their predictive value is inconclusive since the analysis was retrospective and no serial measurement was performed (Table 7).

## Future challenges and limitations

**Figure 1:**
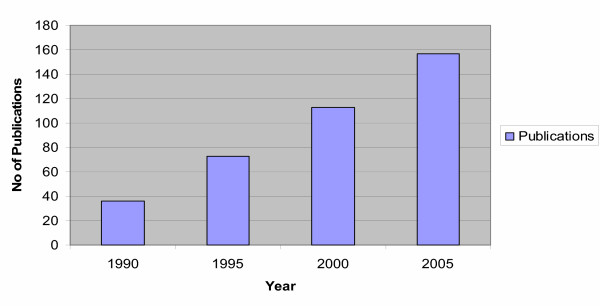
Diagram showing the number of published papers regarding serum biomarkers in patients with ILDs the last fifteen years.

ILDs represent a diverse group of lung diseases comprising over 200 different members that have been broadly classified into several categories [114]. IPF accounting for 46% of all ILD diagnoses in men and women [124] is rapidly becoming one of the most lethal non-malignant lung disorders in the Western world with incidence that overcomes 30 new cases per 100.000 persons per year and with mean survival 3–5 years regardless of treatment [47]. On the other hand several patients with sarcoidosis develop irreversible lung damage and pulmonary fibrosis which culminates to a fatal outcome. The past 15 years, their devastating incidence and clinical seriousness have stimulated innumerable research studies, with any possible approach (Figure 1). Currently, the most fruitful application is monitoring the disease activity and consequently the early detection of patients with increased likelihood of non-response to treatment and progressive disease. Nevertheless, there are problems with the sensitivity, effort-dependability and mainly the ease of repetition of the current modalities being used for this purpose, including BAL, pulmonary function tests and HRCT.

Therefore, a major challenge would be the determination of a reliable serologic marker reflecting the disease behaviour before it becomes obvious in clinical level, easily reproducible and feasible to be measured serially, meaning to be acceptable by the patient. The early serial measurement of this biomarker may lead to an early detection of relapse and thereby allow anti-inflammatory and other treatments to be evaluated or eventually modified before they have failed. The latter components can potentially compile a clinician's "wish list". On the basis of this conception, an important number of serum markers either lung-specific proteins and cancer-associated antigens or cytokines and other serological parameters probing different aspects of the immunopathogenesis of ILDs has been delineated. Initial reports of these markers shed further light into the pathophysiology of ILDs, [75,107,108,119,120,122] improved diagnostic and prognostic capabilities [55-58,61,74,107-111] and ultimately, led to a better patient care. Although the applications of these markers in the clinical setting created major expectations, however the feeling of excitement comes in contrast with important deficiencies exhibited by the new methodologies including non-standardization techniques, lack of knowledge of reproducibility and link to disease behaviour. Moreover, most of the studies enrolled a limited number of patients, insufficient to extract any meaningful or statistically significant outcome. In addition, it should be noted that many of the caveats arising from these findings are generated by the origin disadvantages of the investigated serological parameters to serve as ILD-specific markers since the most of them are influenced by malignancies [12,34,35,53], inflammatory diseases [54], or other lung disorders [26,121]. Furthermore, although the majority of them showed a moderate to high clinical utility as detectors of lung disease and correlated relatively well with the disease severity, however they failed to serve as reliable prognosticators of decline or responsiveness to therapy whereas evidence derived from the evaluation of their histospecific diagnostic accuracy is scarce and limited [61] (Table 8). Additionally, the elevation of biomarkers designed to reflect disease severity is likely to mirror not only the increased rate of entry into the circulation across the damaged air-blood barrier, but also the decreased clearance resulting from significant renal dysfunction seen in ILD patients [120,121]. Finally, it is of high importance to note that unfortunately only the minority of the studies [42,51,55,56,65,109,122] clarified the effectiveness and the diagnostic accuracy of the biomarkers by applying ROC curve analysis which is essential to estimate the sensitivity and specificity of a marker and moreover to define its discriminative cut-off levels. The aforementioned observations coupled with the evidence that the pathogenesis or even the definitions of ILDs and mainly IPF still remain poorly understood and ambiguous and therefore the role of all these molecules associated with their pathogenetic mechanisms has not been yet well determined, may explain the several limitations in the clinical use of serum markers.

**Table 8:** Scoring of various clinical utilities for key biomarkers in ILDs

**Clinical Utilities**	**Key Biomarkers**						
	KL-6	SP-A	SP-D	CC16	sIL-2R	ACE	TNFa

Detection of lung disease	+ +	+ +	+ +	+	NE	+ +	+ +
Histospecific diagnostic accuracy	+ / -	+	+ / -	NE	NE	+	NE
Correlation with disease severity	+ +	+	+ +	+	+ +	+	+ +
Prediction of response to therapy	+	+ / -	+ / -	NE	NE	+ / -	NE
Prediction of decline	+	+ / -	+	+ / -	+	+ / -	+ / -

Abbreviations: ACE: Angiotensin Converting Enzyme, CC16: Clara-cell protein 16, KL-6: Krebs von den Lungen-6, LDH: Lactate Dehydrogenase, ILDs: Interstitial Lung Diseases, ILDs: Interstitial Lung Diseases, sIL-2R: soluble interleukin-2 receptor, SP: Surfactant Protein, TNF: Tumor Necrosis Factor NE: Not Evaluated, 0: No utility, + / -: Low, +: Moderate, + +: High Utility

Furthermore, the application of biological markers in the research field relevant to occupational and environmental lung disorders have raised some additional concerns. Most of the studies exhibited major limitations including the retrospective analysis, the heterogeneity of the studied population and the lack of serial measurements necessary for the adjustment to disease behaviour and the link to disease prognosis. The only so far follow-up study [83] identified cytokine release as a reliable prognosticator of the disease progression. For biomarkers to become a useful addition to the array of tools for investigating occupational and environmental lung diseases appropriate and carefully designed study populations together with interdisciplinary collaborations and financial supports are required. Nevertheless, ethical, legal and social issues of human experimentation that arise with the application of biomarkers need to be considered prior to conducting studies [78].

Collectively, these findings highlight the necessity for further validation prospective studies and the assessment of novel molecules [75] to serve as diagnostic and prognostic tools as well as markers of the disease activity and severity. The recent application of massive genome screening tools such as DNA microarrays in the respiratory research field [125] has led to an increase rate of discovery of genes involved in the disease initiation and progression. Thereby, the next challenge arising from the emerging of hundreds of candidate biological markers is the application of the genome discoveries in the clinical setting with the use of tissue microarrays [126] in order to establish their diagnostic, prognostic and therapeutic importance and lead to a better understanding of the biological characteristics of ILDs.

## Conclusion

Currently, the application status for most of these biologic markers is still in its infancy and remains exploratory. Unfortunately, they do not yield indications for therapy or mark the end of the inflammatory process and their prognostic value still needs to be established. Although the majority of them have not yet lived up to the "great hype" that was generated, lung-epithelium specific proteins and mostly KL-6 and SP-D show the greatest promise in IPF and other ILDs whereas serum cytokines seem to be not ready for routine monitoring. As for sarcoidosis, despite the controversial aspects regarding the usefulness of ACE in reflecting the disease activity, it still remains the most reliable and well defined marker whereas sIL-2R represents one of the most fruitful applications. Finally, in occupational and environmental lung disorders the only so far follow-up study supports TNF release as a valuable predictor of the fibrotic disease progression, outcome and activity (Table 8).

Further prospective investigations, technical improvements and introduction of novel markers are warranted in order to elevate the association of serum biomarkers with the pathogenesis of ILDs in the same status as for tumour markers with lung cancer. Nonetheless, crossing the boundary from research to clinical application requires validation in multiple settings, experimental evidence supporting a pathophysiologic role, and ideally intervention trials showing that modification improves the outcome. The emergence of pioneering technologies including DNA and tissue microarrays which have already been applied with great success in the respiratory research field can help scientists to circumvent this problem and bridge this boundary. In the interim, these markers can be quite useful to supplement the clinical, radiological and physiological monitoring of the disease and identify high-risk patients who would benefit from aggressive management of established risk factors.

## List of Abbreviations

Angiotensin converting enzyme (ACE)

Bronchoalveolar lavage (BAL)

Carbohydrate antigen sialyl Lewis a (Ca 19-9)

Chronic beryllium disease (CBD)

Clara Cell Protein (CC16)

Collagen vascular disease-associated interstitial pneumonia (CVD-IP)

Cytokeratin fragment 19 (CK19)

Coal Worker's Pneumoconiosis (CWP)

Enzyme-linked immunosorbent assays (ELISA)

Extracellular matrix (ECM)

Farmer's lung disease (FLD)

High resolution computed tomography (HRCT)

Idiopathic pulmonary fibrosis (IPF)

Interferon-γ (IFN-γ)

IFN-inducible T cell-a chemoattractant (ITAC)

Interstitial lung diseases (ILDs)

Krebs von den Lungen-(KL)-6

Lactate Dehydrogenase (LDH)

Monocyte chemoattractant protein-(MCP)-1

Monocyte inflammatory protein- (MIP)-1a

Non-specific interstitial pneumonia (NSIP)

Progressive Massive Fibrosis (PMF)

Pulmonary Function Test (PFT)

Receiver-operating-characteristic (ROC)

Sialyl Lewis x (SLX)

Soluble IL-2 receptor (sIL-2R)

Surfactant protein-(SP)

Systemic sclerosis (SSc)

Tumor Necrosis Factor (TNF)

Usual interstitial pneumonia (UIP)

## Competing interests

The author(s) declare that they have no competing interests.

## Authors' contributions

AT and DB were involved with the study conception. AT, GK and SA performed the data acquisition and interpretation. AT prepared the manuscript. DB was involved in revising the article for important intellectual content. All authors read and approved the final manuscript.
